# Silence is golden? Relationship between silent behavior among online community members and operation performance from the perspective of personality trait

**DOI:** 10.3389/fpsyg.2022.912511

**Published:** 2022-08-25

**Authors:** Xueliang Pei, Fanying Lyu, Xiaojun Xiong, Anpin Wei, Jianing Guo, Wenxin Zhou

**Affiliations:** ^1^College of Business Administration, Huaqiao University, Quanzhou, China; ^2^East Business Management Research Center, Huaqiao University, Quanzhou, China; ^3^Business School, University of Leeds, Leeds, United Kingdom; ^4^Department of Business Management, National Taichung University of Science and Technology, Taichung, Taiwan; ^5^School of Management, Wuhan University of Technology, Wuhan, China; ^6^School of Management, Shanghai University, Shanghai, China

**Keywords:** online community, proactive personality, silent behavior, online community identification, operation performance of the online community

## Abstract

As companies are transforming their branding, marketing, operations, and research and development (R&D) by running online communities to build their core competitive advantages in the digital era, the silent majority is still the norm in the online community and has become the focus of online community operations. Thus, it has become the core issue that why silent behavior of online community members occurs and its impact on operation performance of the online community. According to the traditional theory of organizational behavior, this study focuses on the theoretical model of the relationship between proactive personality, silent behavior of online community members (acquiescent, defensive, and prosocial silence), and operation performance of the online community, and further analyzes the impact of community identification on these relationships. Eight hundred online community members in China participated in this study. The results indicate that: (1) proactive personality has a significant negative impact on acquiescent silent and defensive silent behavior of the online community members, and a significant positive impact on prosocial silent behavior of the online community members; (2) The acquiescent silence and defensive silence have a significant negative impact on online community operation performance, whereas prosocial silence has a significant positive impact on community operation performance; (3) The acquiescent silence and defensive silence have a significant mediating effect on the relationship between proactive personality and community operation performance; (4) Online community identification has a moderating effect on the relationship between silent behavior and online community operation performance. The study proposes the mechanisms and double-edged sword effects of the silent behavior of online community members from the perspective of personality traits. On the one hand, it generalizes the research of traditional organizational silent behavior theory to the context of the online community. On the other hand, it provides reference and inspiration for the theoretical research and practical management of silent behavior of online community members.

## Introduction

In recent years, the formation of communities has become a particularly evident trend in the digital era, and online communities have become the main form of contemporary social activities. The emergence of the Internet, especially social media, has facilitated social interaction between users. Such interaction is no longer constrained by geographical distance but has become more network-and community-based (Tanis, 2008). Simultaneously, with the development of social media platforms, the number of online communities has exploded, providing users with a virtual space where community members can build social relationships and experience a sense of belonging similar to real-life (Ertas et al., 2020).

As a result, companies are also responding to this trend, transforming their branding, marketing, operations, and research and development (R&D) by running online communities to innovate the way they create and realize business value and, thus, build their core competitive advantages in the digital era (Farhoomand and Ng, 2003). In recent years, more and more scholars have begun to study the impact of social media and its network on business value creation, and they believed that the core of business value creation is the community established through social media platform (Quinton and Wilson, 2016). At this level, LinkedIn can be regarded as successful businesses practice that online community platform affects the value of enterprises. As the world’s largest professional business social media platform, LinkedIn has 774 million members all over the world, and has established more than 2 million groups in the whole industry, covering more than 200 countries and regions around the world (LinkedIn, 2022). LinkedIn’s group function allows each member to create, manage and join a group related to his major or interest. This group based on members’ active choice and common interests may bring benefits to members or their organizations (Quinton and Wilson, 2016). In fact, LinkedIn can also be regarded as a huge online business community, where all members of LinkedIn’s community build connections with others through knowledge sharing. This connection can not only bring benefits to the participants, but also improve the economic value and performance of the enterprises behind the participants (Stephen and Toubia, 2010).

But not all online communities have such large-scale community members and active community activities as LinkedIn. A common and typical phenomenon encountered by many online community operators is that the majority of online community members exhibit silent behavior, which makes social interaction in online communities fail to meet expectations and causes problems for the sustainable operation of online communities. Wikipedia, which was established 2 years before LinkedIn, is facing the crisis of community decline. As the largest encyclopedia online community in the world, the number of posts on Wikipedia has been decreasing in recent years, and the content contributors have been shrinking (Frick, 2013). Since 2005, the number of editors of wikipedia has been declining. Moreover, due to the problems of community management system and community environment, not only are the existing active editors decreasing, but the attraction of Wikipedia to new editors is also decreasing. Like most online communities, Wikipedia relies on a small number of editors to do a lot of editing (Mandiberg, 2020). This decline in participation has had a negative impact on Wikipedia’s performance to a certain extent, that is, the quality of many articles on Wikipedia can’t reach the standard, and most of them can’t even reach the intermediate quality standard set by Wikipedia itself (Simonite, 2013). To improve editor loyalty, Wikipedia Foundation’s research mainly focuses on the participation motivation of active editors, while ignoring most silent editors (Mandiberg, 2020). In fact, Katz (1998) and Mason (1999) revealed that 90% of online community members are in a silent state, which makes it impossible for the operation of communities to reach their desired goals. Therefore, understanding the reasons for the silent behavior of online community members and finding effective ways to reduce such behavior has become a key topic of concern for online community operators.

Traditional research on organizational behavior has also focused on the phenomenon of organizational silence. Morrison and Milliken (2000) argued that the silent majority exists in any organization and is a collective phenomenon in organizations. Scholars have conducted several studies on employee silent behavior in organizations, exploring the dimensions, factors, and consequences of organizational silence. For example, Dyne et al. (2003) conceptualized employee silence and categorized it into three types, namely acquiescent silence, defensive silence, and prosocial silence. Khalid and Ahmed (2016) analyzed the effect of perceived organizational politics and supervisor trust on employee silence. ParlarKılıç et al. (2021) studied the effect of organizational silence on job satisfaction and performance levels. These studies are all studies on silent behavior in traditional organizational contexts.

Nonetheless, unlike traditional organizations, online communities have unique characteristics in that members are authorized to communicate, interact, and collaborate equally anytime, anywhere, and without the constraints of physical and traditional organizational structures (Zablith et al., 2016). Accordingly, the silent behavior of online community members differs significantly from the silent behavior in traditional organizations, and its concepts, factors, and consequences await further research (Luarn and Hsieh, 2014). Accordingly, following the framework of research on silent behavior in traditional organizations, we propose the following research questions in this paper: (1) How does silent behavior in traditional organizations change in the context of online communities? (2) Why does silent behavior arise in online communities? (3) What impact does silent behavior in online communities have on the operation performance of online communities?

Responding to the first two questions, this paper’s focus was on community members (the subjects of online communities) to examine the definition and factors of their silent behavior. This approach differs from previous research on silent behavior in traditional organizations that focus on the impact of organizational culture, organizational politics, and leadership behavior on organizational silence (Dedahanov and Rhee, 2015; Dedahanov et al., 2016; Gencer et al., 2021). Given the informal organization of online communities and the spontaneous nature of members’ behavior (Tsai and Bagozzi, 2014; Kaur et al., 2019), this paper examines the mechanism of silent behavior among online community members from the perspective of members’ personality traits and focuses on whether and how personality traits in the real world affect their silent behavior in online communities.

As for the third research question, how to improve the operation performance of online communities is a core issue for online community operators (Fan and Janssen, 2021; Pu, 2021). Subsequently, this study also aims to investigate ways to improve the operation performance of online communities by guiding, supporting, and operating based on the silent behavior of community members. Accordingly, this paper focuses on the impact of members’ silent behavior on the operation performance of an online community.

Finally, regarding the online community, this paper draws on the traditional research on organizational silence (Dedahanov et al., 2016; Gencer et al., 2021), and introduces the concept of online community identification to investigate its impact on the relationship between personality traits, the silent behavior of community members, and operation performance of online communities.

## Literature review

### Basic concepts

#### Online community

Online communities are online social groups based on digital technologies, where users can create, edit, and comment on the content of interest and communicate and interact with each other in a virtual space at any time (Majchrzak et al., 2013). Preece’s (2001), p. 348 definition of the online community can support this opinion, “any virtual social space where people come together to get and give support, to learn, or to find company.” Online communities based on social media have broken geographical boundaries in social interactions among netizens and are characterized by networks and clusters. Lately, the commercialization of online communities has attracted more and more attention, and scholars have found that online communities can not only provide a platform for knowledge sharing, emotional support, and entertainment but also facilitate business activities (Phang et al., 2009). Because of the timeliness of online community information response, enterprises can improve organizational performance through online community management (Lee et al., 2022). Further, online communities can bring significant business benefits to online community operators through knowledge seeking and sharing, act as hubs for social and information networks, and generate platform effect (De Valck et al., 2009). As a result, online communities have now evolved from purely spontaneous communities of netizens to a modular ecology of online communities with each community displaying distinctive features.

This paper considers online communities as Internet-based social groups formed by netizens with the same values, interests, or benefits who are connected through social media platforms. In comparison to traditional offline communities, the mode of information dissemination in online communities is based on the network structure of digital technologies and is characterized by high speed, large influence, a great number of people involved, and the possibility of infinite expansion beyond temporal or spatial boundaries.

#### Proactive personality

This paper introduces proactive personality as a major contributing factor to the silent behavior of online community members from the perspective of personality traits. In the research on aggressive behavior, Bryce et al. (2021) mentioned that different personality traits can help predict people’s aggressive behavior. Therefore, this paper holds that the level of individual’s proactive personality can also help us to recognize the silent behavioral logic behind it. Bateman and Crant (1993) introduced the concept of proactive personality and argued that people with a proactive personality have a comparatively stable behavioral tendency to enact changes in their surrounding environment. On this basis, Fuller and Marler (2009) put forward “Proactive personality is an individual’s dispositional tendency to alter their environment through proactive behavior.” Grant and Ashford (2008) also pointed out that being proactive means that rather than watching things happen, one chooses to actively control where things are going. Research has demonstrated that people with high proactivity will aspire and make efforts to change their environment or themselves to achieve a different future. For online communities, the operation performance of an online community depends on the active performance of community members, and their ability to take initiative and actively integrate into the surrounding environment. These factors are crucial to community members’ behavior and to improve the operation performance of online communities. Therefore, this paper analyzes the relationship between proactive personality, silent behavior in online communities, and operation performance of online communities from the perspective of proactive personality. Therefore, this paper starts with the realistic attributes of personality traits, and analyzes the relationship between proactive personality, silent behavior in online communities, and operation performance of online communities from the perspective of proactive personality.

#### Silent behavior

The research on silent behavior originated in the field of organizational behavior. Morrison and Milliken (2000) introduced the concept of organizational silence, “We argue that there are powerful forces in many organizations that cause widespread withholding of information about potential problems or issues by employees. We refer to this collective phenomenon as organizational silence,” which they considered a collective phenomenon in organizations where employees are silent because they are concerned about making negative statements, or they believe that their statements are not important to the organization. Pinder and Harlos (2001) perceived organizational silence as employees’ withholding their evaluations of an organization although they are capable of improving current organizational performance. Dyne et al. (2003) deemed organizational silence as the purposeful silence of employees on ideas, information, and opinions that may improve an organization.

When it comes to online communities, the famous 90-9-1 rule states that in websites adopting the online community model, 90% of participants only consume content, 9% of the participants actively change or update content, and only 1% of the participants actively produce new content (Arthur, 2006). Therefore, in the research of online community participation, scholars put forward the concept of “Lurker.” “Lurker” usually refers to people who observe silently but do not participate in activities or remain silent in online communities (Edelmann, 2013). And lurkers’ behavior in online community is called “lurking.” Generally speaking, lurking is associated with non-public participation, inactive and silent (Leshed, 2005; Nonnecke et al., 2006). Preece et al. (2004) also pointed out that, in some online communities, more than 90% of the members are lurkers. They do not express their opinions and focus only on browsing information. Thus, it can be argued that silent behavior in online communities is an ever-present norm that needs further study.

This paper defines and measures the silent behavior of online community members by, primarily, drawing on previous studies of organizational silence. Pinder and Harlos (2001) first categorized different dimensions of employee silence, classifying employee silence into employee quiescence and employee acquiescence. Acquiescence signifies that employees passively withhold their opinions and conform to the *status quo* due to feeling incapable to make changes. Quiescence refers to employees’ intentional omission of their opinions out of the need to protect themselves or maintain interpersonal relationships. Based on Pinder and Harlos (2001) and Dyne et al. (2003) further differentiated three types of employee silence: acquiescent silence, defensive silence, and prosocial silence. Among them, prosocial silence means employees intentionally choose to be silent out of altruism or collaboration, thus, its starting point is different from that of the first two types of silence. In this paper, we refer to Dyne et al. (2003) for the definition and differentiation of silent behavior.

### Relationship between members’ proactive personality and operation performance of online community

In organizations, Bateman and Crant (1993) argued that proactive personality reflects a comparatively stable tendency of individuals to proactively control their external environment and actively initiate changes. Rather than simply reacting to their environment, people with high proactivity look for opportunities to act proactively in an ever-fluctuating environment. They tend to play a leading role in the development of an organization, influencing the organizational environment through their personal development while achieving better personal performance. Some studies also suggested that to enhance their performance, people with high proactivity actively choose and even create environments that are conducive to obtaining higher levels of performance (Crant, 1995). Accordingly, employees with proactive personalities tend to perform better than those without because their participation can speed up the advancement of an organization’s tasks to a large extent (Crant, 1995).

In studies on online communities, scholars have focused on opinion leaders as representatives of people with high proactivity. Opinion leaders influence the surrounding environment for public opinion through their proactive output of opinions. Further, they lead the development of social opinions while permitting more opinions to be presented, thus increasing community activity (Lin et al., 2018; Tobon and Garcia-Madariaga, 2021). Therefore, in online communities, members with high proactivity participate more actively in the activities of the community and exhibit more organizational citizenship behaviors. These behaviors will spur the behaviors of other members to a certain extent and may create a positive community atmosphere that allows communities to improve operational performance. Therefore, we propose the following hypothesis:

H1: Online community members with proactive personalities positively influence member performance and community performance of online communities.

### Relationship between proactive personality and silent behavior of members of online community

In traditional research on organizational behavior, researchers have argued that people with high proactivity tend to take more active actions, such as breaking organizational silence and voicing opinions and suggestions. Empirical studies have indicated that proactive personality is one of the key factors in the willingness of employees in organizations to speak up (Morrison et al., 2015). Fuller and Marler (2009) also found, through meta-analysis, that an individual’s proactive personality has a significant impact on their organizational citizenship behavior, relationship-building behavior, innovation behavior, and other proactive and positive behaviors. Further research shows that members with high proactive personalities in the organization will bring better performance to the organization by putting forward new ideas, actively innovating, or achieving higher productivity, etc. (Baer and Frese, 2003). Hence, the level of members’ proactive personality behavior has an overall effect on organizations. Members with high proactivity have the higher organizational commitment and are more willing to give input that facilitates organizations achieving higher performance goals.

In online communities, proactive personalities can drive members to actively participate in community activities, breaking the silence, and demonstrating more organizational citizenship behaviors. In contrast, members with low proactivity display passive adaptation to environmental changes, fail to recognize external opportunities, and therefore tend to be more comfortable with retaining the *status quo*. They can be described as having a “just getting by” attitude (Zhang et al., 2012). According to existent research, acquiescent silence refers to members’ negative withholding of views and passive conformity to the current environment. Low proactivity leads to a passive attitude, which gradually marginalizes members’ presence in the community and eventually leads to acquiescent silence.

Members with high proactivity have positive and enterprising qualities and are more willing to engage with organizations and groups. Generally speaking, individuals with high proactivity will actively seek recognition and help from other members of the community, actively integrate into the community, and build their social network within the community, thereby gaining more social capital, thus, realizing their full potential. This is because people with high proactivity tend to take the initiative to create favorable conditions for success in their personal development (Li et al., 2010). In other words, members with high proactivity actively seek to establish favorable interpersonal and supportive relationships within the community to boost their achievement. For example, studies have indicated that employees with high proactivity within organizations build high-quality exchange relationships with their supervisors which support their development (Li et al., 2010). From this perspective, high proactivity has an inhibiting effect on defensive silent behavior that derives from the need to maintain interpersonal relationships.

In the interim, similar to findings on traditional organizational behavior, online community members with high proactivity are more likely to adopt prosocial silent behavior to evade negative effects when they realize that their behaviors within the community may negatively affect other community members. Accordingly, they are more willing to engage in prosocial behavior that benefits the community and its members, thus facilitating the operation of online communities.

Accordingly, we propose the following hypotheses:

H2: The proactivity of online community members negatively affects the acquiescent silent behavior and defensive silent behavior of community members.

H3: The proactivity of online community members positively affects the prosocial silent behavior of community members.

### Relationship between silent behavior of community members and operation performance of online community

Operation performance refers to the measurable aspects of the outcomes of a company’s operations, which are generally measured by objective output indicators (Trienekens et al., 2005). Existing studies have classified the operation performance of online communities into two levels: group level and individual level. The group level of operational performance can be assessed about indicators such as the company’s performance, brand equity, and innovation capability, while the individual level of operating performance of an online community can be evaluated by drawing on the concept of “engagement” in marketing, community members’ satisfaction, self-brand connection, brand loyalty, etc. as the basis for judgment (Hollebeek et al., 2014). Hajli et al. (2015) argue that communities will cease to exist when there is no sustained engagement from community members. In the research of marketing on customer integration, consumers’ silence is generally due to reasons such as out of control or inability to speak. For enterprises, consumer silence is harmful, because enterprises don’t know why consumers are dissatisfied and have no ability to prevent consumers from leaving (Schaarschmidt et al., 2021). From this perspective, the impact of consumer silence on enterprises is similar to that of online community silence on online community. Therefore, for online community operators who want to enhance their operational performance, reducing the silent majority within the community and increasing community members’ continuous engagement is the key to the survival of the community. At the group level, the growth of the organization, the more frequent and stable the interaction between community members, and the more social capital the community accumulates, the higher the operation performance of the community (Lesser and Storck, 2001).

Previous studies on the operation performance of online communities have primarily focused on the individual level, but we argue that measuring the operating performance of online communities on the individual level only is insufficient. To measure the operating performance of online communities more comprehensively, not only do we analyze the operation performance at the individual and group level, but we also distinguish operation performance into community performance and member performance. Drawing on previous research, we propose that the operation performance of online communities on the individual level can be measured in terms of community members’ willingness to share, satisfaction with the community, and evaluation of the community. While the group level can be measured in terms of community activity, community size, community membership turnover rate, and community impact.

According to organizational identity research, Morrison and Milliken (2000) argued that the negative impact of organizational silence on decision-making and change processes in organizations increases as the level of pluralism within organizations rises. Organizational silence is particularly detrimental in rapidly changing environments. In this context, organizational silence prevents high-level managers from obtaining accurate internal feedback, which in turn affects organizational performance. Harlos and Pinder (2000) argued that employee silence stems from the unmet needs for a sense of belonging and safety within an organization and low levels of psychological security among employees. These unmet needs deplete employees’ physical and psychological resources, resulting in emotional burnout and behaviors of psychological and physiological withdrawal. Regarding online communities, defensive silent behavior based on self-protection reduces community members’ active participation, hinders the operator’s control over the community members’ circumstances and understanding of their ideas, reduces activity, and, thus, negatively affects the operation performance. According to the research on “Lurker,” the existence of lurkers will lead to the fading of the group, because the active participants in the group can’t continue the discussion or will feel depressed because they can’t get the support and feedback from most silent members in the group (Ping and Chee, 2001). The success of online communities and online tools depends on the active participation and contribution of members, while lurkers’ silence can be regarded as a blow to the sustainable development of the team (Preece, 2004).

From the community members’ perspective, acquiescent silence can lead them to believe that their opinions are not important to the community, which is a form of self-marginalization. Further, community members may experience cognitive dissonance with the community, which may result in their blocking or withdrawing from the community, which in turn negatively impacts the operation performance of the online community.

Nevertheless, the benefits of community members’ prosocial silent behavior significantly contribute to its operation performance. In the research of online community’s lurking behavior, some studies have pointed out that lurking involves a series of complex behaviors and motivations just like other online behaviors. Some lurkers are purely free-riders, but others may have other reasons, including pro-social and altruistic motives (Edelmann, 2013). Lurking with this motivation is similar to pro-social silence in the organization. If we look at lurkers from a positive perspective, they can also be regarded as effective participants in the community and can support the innovative activities of the online community. Considering that lurking is the most common behavior in online community, lurkers actually spend a lot of time in the community to get acquainted with others silently and accumulate knowledge about the community, even if they seem to be in a state of silence (Edelmann, 2013). Therefore, there is an opinion that lurkers can change from invisible members to visible members in the future, providing key source of revenue or important information for community development (Ostrom, 1990; Ridings et al., 2006). From this point of view, the influence of lurkers’ silence on community performance is not only negative influence, but there may also be positive influences. Therefore, we propose the following hypotheses:

H4: The acquiescent silent behavior and defensive silent behavior of online community members negatively affect both community performance and member performance.

H5: The prosocial silent behavior of online community members positively affects the operation performance of online communities.

### Relationship between community members’ proactive personality, silent behavior and operation performance of online community

In traditional research on organizational behavior, according to Hogan and Holland’s (2003) model of the job-performance relationship, personality affects job performance through work behavior, and work behavior plays a mediating role between personality and performance. In organizations, employee silence is defined as the intentional withholding of ideas, suggestions, or concerns about work issues. It is an employee’s conscious effort to inhibit their actions based on whether they believe their actions can make a difference (Guenter et al., 2017). According to Dyne et al. (2003), employees choose to remain silent for various reasons, such as protecting themselves from feeling attacked, being comfortable with the existing organizational pattern, or protecting organizational secrets. In most cases, employee silence is considered a contributing important factor to several negative outcomes in organizations, for instance, low employee satisfaction or poor job performance (Brinsfield et al., 2009). However, some studies have shown that silent behavior may also promote the development of operation performance of online community under the action of proactive personality. Willett and Lynch (1998); Takahashi et al. (2003), and Takahashi et al. (2007) put forward the concepts of active lurkers and passive lurkers, in which active lurkers are those who directly contact with posters or spread information outside the online community, while passive lurkers only silently read the content they need. Therefore, active lurkers may enhance the influence of their online community or bring new contacts and members by transmitting information to the external environment (Edelmann, 2013).

As an online form of organization, the operation performance of online communities is important, but often, operators find it difficult to increase community activity. For operators, strengthening the management of online communities has a positive impact on the improvement of operational performance, but this effect is lower than that of traditional organizations. As a result, focusing on the community members themselves may be the best way to resolve the predicament. According to behavioral plasticity theory, individuals respond to social factors differently (Brockner, 1983). Behavioral plasticity theory assumes that an individual’s self-esteem is a prerequisite for their behavior. It predicts that people with low self-esteem are more likely to be influenced by social situations than people with high self-esteem (Brockner, 1988), where social situations include peers’ behavior in the work environment, the leadership of superiors, etc. (LePine and Van Dyne, 1998). Although self-esteem does not equate to proactive personality, proactive personality characterizes those who transcend situational constraints and in turn influence their environment (Bateman and Crant, 1993, p. 105). Just like individuals with high self-esteem, individuals with higher proactivity are less likely to be influenced by their environment, especially compared to individuals with lower proactivity (Guenter et al., 2017). Individuals with high proactivity are self-determined, thus, they are more inclined to take positive actions to change their existing environment (Seibert et al., 1999). Therefore, we argue that behavioral plasticity theory can also be applied to explain how proactive personalities in online communities influences members’ online silent behavior and, ultimately, change the operation performance. Once the silence in an online community is broken, the activity increases greatly, therefore, improving performance. Subsequently, we propose the following hypothesis:

H6: Online community members’ silent behavior mediates the relationship between proactive personality and community performance and member performance.

### The role of online community identification

According to social identity theory, people tend to define themselves per the group they belong to and they strive to improve the status of their group due to a sense of belonging. Social identity theory explains an individual’s identification with other members of a community (Bagozzi and Dholakia, 2002). There are many studies on online community identification. Hogg and Abrams (1988) stated that members build social identifications according to their sense of belonging to the group and the extent to which they derive benefits from social interactions. When members identify themselves as part of an online community, they are more likely to join and actively participate in the activities of the community (Dholakia and Bagozzi, 2004).

In current research on social identity in virtual communities, scholars have proposed that social identification can be divided into three aspects: cognitive identification, affective identification, and evaluative identification (Ellemers et al., 1999; Bergami and Bagozzi, 2000). Ellemers et al. (1999) distinguished the three aspects theoretically. Bergami and Bagozzi (2000) built on Ellemers et al. (1999) and further divided the affective aspect into the positive feelings a person receives from the organization (e.g., enjoyment, happiness) and the feelings (e.g., attachment, belonging) a person has toward the organization. They suggested that the cognitive, emotional, and evaluative aspects can not only be measured as components in social identity but also influence people’s tendency to behave in the organization. Drawing on these three aspects, this paper classifies online community identification into cognitive online community identification, affective online community identification, and evaluative online community identification.

Online community identification is the sense of identity that community members gain from belonging to an online community. This sense of identity may motivate online community members to adopt positive behaviors that are beneficial to the development of the community. Specifically, for example, players in an online gaming community may join a particular gaming gang, take pride in being a member of that gang, and all their actions in the game revolve around defending the honor of the gang. Moreover, in online brand communities such as “Apple fans,” “MI fans,” and others, members have a strong sense of “fanship” and identify themselves as loyal fans of a brand. Not only do they pay close attention to the development of the brand, but also defend the brand’s reputation. The logic of this behavior is that online community members psychologically classify themselves as “insiders” of a community, and this sense of identity urges community members to subconsciously reduce behaviors (such as silent behavior) that are detrimental to the development of the community. Rather, they tend to boost the development of the community through their actions, given that “benefit to one means benefit to all, whereas harm to one means harm to all.” According to Morrison and Milliken (2000), employees in organizations form shared perceptions through informal social networks which amplify and increase the likelihood of forming a strong atmosphere of silence. This is especially true when there are structural and managerial factors present in the organization that are conducive to organizational silence. According to social identity theory, community identification is the process of members’ self-categorization, by which members gain a sense of identity as they psychologically believe they belong to the community (Stets and Burke, 2000). Automatically, this sense of collective identity influences members’ participation (Muniz and Schau, 2005). Kang et al. (2014) study on restaurant fan communities on Facebook indicated that social-psychosocial benefits have a positive impact on members’ active participation in online communities, where the social-psychosocial significance includes members’ identification with the values of the restaurant. Further, community identification can help members identify with a group and build emotional bonds. For example, employees with high community identification are more likely to associate themselves with their organization, and they tend to reduce silent behavior and show stronger organizational commitment and more organizational citizenship behaviors (Bergami and Bagozzi, 2000), which contributes to the improvement of community performance. Therefore, we argue that the higher the online community identification, the fewer online communities and operation performance are negatively impacted by silent behavior. Based on this, we propose the following hypotheses:

H7: Online community identification moderates the relationship between acquiescent silent behavior, defensive silent behavior, and operation performance of online communities. High online community identification weakens the negative effects of acquiescent and defensive silent behavior on the operation performance of online communities.

H8: Online community identification moderates the relationship between prosocial silent behavior and operation performance of online communities. High online community identification enhances the positive effect of prosocial silence on the operation performance of online communities.

In an organizational environment, the organization that the individual identifies with is the key context to activate proactive personalities (Wu et al., 2021). Online community identification reflects self-congruity between members and the community, thus, when proactive members identify with the values of the community, they see themselves as part of the community and representatives of the whole. Proactive members with a strong online community identification will think and act in terms of the group’s goals and interests and will actively participate so long as it is beneficial to the community (Shi and Wang, 2014), thus positively influencing the operation performance of the community. Conversely, when members do not identify with the community they are in and do not share a high sense of belonging to the community, members will stray from activities. This is true even for members with high proactivity. Without community identification, they will lose the willingness to actively participate in activities, making it impossible for community operators to improve the operation performance of the online community. Based on this, we propose the following hypotheses:

H9: Online community identification moderates the relationship between proactive personality and operation performance of online communities. While high online community identification enhances the positive effect of proactivity personality on operations performance of online communities.

### Theoretical model

**FIGURE 1:**
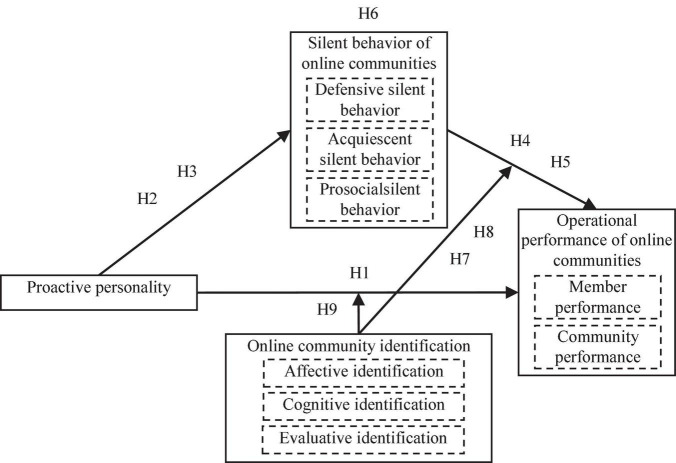
Research model.

Based on social identity theory, this paper analyzes the influence of proactive personality on the operation performance of online communities and constructs a conceptual model of the relationship between proactive personality, community members’ silent behavior, and the operation performance of online communities. The theoretical model of this study is shown in Figure 1. Nonetheless, due to differences in the levels of proactive personality, the defensive silent behavior of community members also varies. Consequently, we insert online community identification as a moderating variable to explore the effect of online community identification on the relationship between the independent and dependent variables.

## Research methods

### Questionnaire survey

This study was conducted in the context of online communities, thus, prominent communities with substantial traffic flow were our focus and where we distributed our questionnaire. The questionnaire was designed using Questionnaire Star (wjx.cn) and distributed in the form of an online questionnaire to online community members with experience participating in online communities. To ensure the reliability and validity of the questionnaire, a pilot survey was conducted in three online communities with high traffic flow, namely WeChat, QQ group, and Douban group, before the formal survey, and the questionnaire was modified according to the problems that surfaced during the pilot survey. A total of 150 surveys were returned from the pilot survey. In response to the feedback from some respondents that the concept of community was not clear to them, an explanation of the concept of community was added to the questionnaire. After the pilot survey, formal data collection was completed on the Credamo platform. To ensure that participants filled out the questionnaire carefully, a pair of opposite questions and a pair of similar questions were included in the questionnaire. They are: “I feel happy every time I enter this community,” “Participating in the activities of this community takes too much energy from me and I feel exhausted,” “I often participate in the activities organized by the community operator” and “I am interested in the activities of the community, and I often interact with the community members.” Self-contradictory answers to these questions were considered invalid responses and the corresponding respondents were eliminated from the analysis. After more than a month of data collection, a total of 800 surveys were returned. Lastly, in the process of questionnaire screening, to maximize the validity of the data, after excluding responses with self-contradictory answers to the pair of opposite questions or the pair of similar questions, we further eliminated157 respondents who completed the survey within less than 180 s from the analysis. The final sample consisted of 643 respondents with an effective response rate of 80.4%.

### Measures

This study, mostly, used the 5-point Likert scale to measure respondents’ various attitudes (1 = completely disagree, 5 = completely agree). In this study, the measurement items of defensive silent behavior in the silent behavior of online communities were adapted from the scale of quiescent silence proposed by Knoll and Van Dick (2013). The measurement items of acquiescent silent behavior referred to the research of Preece et al. (2004), while the measurement items of prosocial silent behavior were inspired by the discussion of the concept of prosocial silence in the research of Dyne et al. (2003). The items setting of the online community identification scale not only referred to the research of Dholakia et al. (2004), which divided the online community identification into three dimensions: cognitive, affective, and evaluative identification but also referred to the organizational identification questionnaire (OIQ) proposed by Miller et al. (2000). The design of the measurement items of community performance referred to the five-item measurement scale of operational performance proposed by Ahmad and Schroeder (2003), and the design of the items of member performance referred to the organizational commitment dimension of intangible performance scale in the article of Ahmad and Schroeder (2003). Finally, the scale of proactive personality adopts the research of Li et al. (2014).

### Descriptive statistical analysis

Descriptive statistics of the sample are shown in Table 1. In this study, the proportion of male respondents was 49.5%, slightly lower than the proportion of female respondents, which was 50.5%. Generally speaking, the gender ratio of the sample was balanced. In terms of age, the sample was dominated by respondents aged 21–30 years old, accounting for56.9%; Those aged 31–40 accounted for 34.2%. The proportion of respondents aged 41–50, 20 years old and below and over 50 years old were smaller, accounting for 4.5, 3.4, and 0.9%, respectively. This largely reflects the age structure of netizens today, as young netizens are the main participants in online communities. In terms of educational background, respondents with undergraduate education accounted for the largest proportion at 72.6%. Regarding whether they have an avatar in the community, 80.6% of the respondents answered positively and 9.6% answered negatively, while 9.8% chose to use the system default when joining online communities. This indicates that online community members prefer to join online communities with an avatar that helps conceal their personal identity rather than using a real image.

**TABLE 1:** Characteristics of the study sample.

Category		Frequency	Percent (%)
Gender	Male	318	49.5%
	Female	325	50.5%
Age	20 and under	22	3.4%
	21–30	366	56.9%
	31–40	220	34.2%
	41–50	29	4.5%
	50 and above	6	0.9%
Educational Background	Undergraduate below	118	18.4%
	Undergraduate	467	72.6%
	Postgraduate	48	7.5%
	Ph.D.	10	1.6%
Avatars	I have avatars	518	80.6%
	I don’t have avatars	62	9.6%
	System default	63	9.8%

### Reliability and validity tests

To assess whether each structural variable was truly measured by the corresponding measures, we used Cronbach’s α to test the reliability of the structural variable measures, as shown in Table 2. The Cronbach’s α values for the 9 latent variables in this study were all greater than the recommended value of greater than or equal to 0.6, which indicated that the internal consistency of each structural variable was high and therefore this study had good reliability.

**TABLE 2:** The results of reliability test.

Variables	Items	Cronbach’s α
Cognitive identification	7	0.77
Affective identification	8	0.80
Evaluative identification	6	0.74
Defensive silent behavior	4	0.86
Acquiescent silent behavior Prosocial silent behavior	5 3	0.85 0.60
Community performance Member performance	5 5	0.79 0.78
Proactive personality	5	0.75

Each questionnaire in this paper was filled by the same subject, and we used Likert’s five-point scale and cross-sectional data, which may lead to common method biases. To test the influence of common method biases on the research results, this study uses Haman’s single-factor test method to conduct exploratory factor analysis on all items and extracts the factors whose eigenvalue is greater than 1. It is found that the cumulative variance explanation of the first factor before rotation is 28%, less than 50%, so it is considered that there is no influence of common method biases (Podsakoff and Organ, 1986).

Next, we further examined the overall model using confirmatory factor analysis (CFA), which showed a good fit between the data and the model (χ2 = 363.35, df = 125, *p* = 0.000, χ2/df = 2.907, CFI = 0.946, NFI = 0.920, IFI = 0.946, RMSEA = 0.050). The factor loadings (λ) for each measured question were between 0.53 and 0.81, all being greater than 0.5 and significant at *p* < 0.001. The construct reliability (CR) values were all greater than 0.7 (see Table 3), indicating good internal consistency for all latent variables. The average variance extracted (AVE) values were all greater than 0.5, indicating that the measures have good explanatory power for the latent variables on average and therefore the latent variables have good construct reliability and validity. When a model has discriminant validity, the correlation coefficients between its latent variables must be smaller than the correlation coefficients within the latent variables. In this study, we used the relationship matrix between the latent variables for an assessment, which showed that the square roots of the average variance extracted-values were all higher than the correlation coefficients between the latent variables, representing good discriminant validity, as shown in Table 3. Table 3 presents the means, standard deviations, and square roots of AVE for each variable, as well as the correlation coefficients between the variables.

**TABLE 3:** The results of validity test.

Variables	Mean	S.D.	CR	AVE	1	2	3	4	5
1, Cognitive identification	3.93	0.58	0.72	0.57	–				
2, Evaluative identification	3.34	0.95	0.71	0.55	0.58	–			
3, Defensive silent behavior	2.78	0.76	0.84	0.60	–0.43	–0.69	–		
4, Operational performance of online communities	3.44	0.82	0.77	0.53	0.38	0.37	–0.17	–	
5, Proactive personality	3.63	0.67	0.75	0.58	0.53	0.67	–0.55	0.43	–

## Results

### Test for the direct effect

To test the hypothesized relationships, we used SPSS 22.0 for model analysis, and Table 4 presents the results of hypothesis testing for the 5 direct effects. Firstly, proactive personality significantly and positively affected member performance and community performance (β = 0.67, *t* = 22.61, *p* < 0.001; β = 0.60, *t* = 19.04, *p* < 0.001), indicating that members in online communities with higher proactivity drive member performance and community performance of online communities. Thus, H1 was supported. Then, regressions were conducted on the effects of proactive personality on acquiescent silent behavior, defensive silent behavior, and prosocial silent behavior, and the results showed that proactive personality was negatively related to acquiescent silent behavior (β = –0.44, *t* = –12.26, *p* < 0.001) and defensive silent behavior (β = –0.30, *t* = –8.03, *p* < 0.001), but the effect of proactive personality on prosocial silent behavior was not significant (β = 0.44, *t* = 1.11, *p* > 0.05), implying that H2 was supported but H3 was not supported. Finally, regressions were conducted on the effect of silent behavior on the operation performance of online communities, and the results showed that the coefficients of each standardized path “acquiescent silent behavior → member performance,” “acquiescent silent behavior → community performance,” “defensive silent behavior → member performance,” “defensive silent behavior → community performance,” “prosocial silent behavior → community performance,” and “prosocial silent behavior → member performance” were –0.43, –0.34, –0.32, –0.28,0.16 and 0.15, respectively, with the *p*-values all < 0.001. This indicated significant differences among the variables and thus H4and H5 were supported.

**TABLE 4:** Standardized direct effect of latent variables.

Paths	β	*t*	*F*	Results
PPsul	0.67	22.61***	511.15***	Support
PPppo	0.60	19.04***	362.35***	Support
PPppor	–0.44	–12.26***	150.24***	Support
PPppor	–0.30	–8.03***	64.53***	Support
PPppor	0.44	1.11	1.24	Not support
ASB su	–0.43	–12.0***	143.90***	Support
ASBpor	–0.34	–9.21***	84.82***	Support
DSBpor	–0.32	–8.61***	74.11***	Support
DSBpor	–0.28	–7.27***	52.79***	Support
PSBpor	0.16	4.02***	16.19***	Support
PSBpor	0.15	3.70***	13.71***	Support

***p < 0.001.

CP, Community Performance; MP, Member Performance; PP, Proactive Personality; ASB, Acquiescent Silent Behavior; DSB, Defensive Silent Behavior; PSB, Prosocial Silent Behavior.

### Test for the mediating effect

We used the process plug-in in SPSS programmed by Hayes (2012) to perform a bootstrap test for the mediation model, and the specific model selected for testing is model 4. The results are shown in Tables 5, 6. Proactive personality had a significant positive predictive effect on member performance and community performance (B*_*member*_* = 0.69, *t_*member*_* = 22.61, *p* < 0.001; B*_*comunity*_* = 0.68, *t_*community*_* = 19.04, *p* < 0.001), and the positive effect of proactive personality on member performance and community performance remained significant when the mediating variable, acquiescent silent behavior, was added (B*_*member*_* = 0.61, *t_*member*_* = 18.46, *p* < 0.001; B*_*comunity*_* = 0.63, *t_*comunity*_* = 15.99, *p* < 0.001). The negative predictive effect of proactive personality on acquiescent silent behavior was significant (B = –0.72, *t* = –12.26, *p* < 0.001), as was the negative predictive effect of acquiescent silent behavior on member performance and community performance (B*_*member*_* = –0.11, *t_*member*_* = –5.31, *p* < 0.001; B*_*comunity*_* = –0.07, *t_*comunity*_* = –2.83, *p* < 0.001). Proactive personality also had a significant positive predictive effect on member performance and community performance when the mediating variable was changed to defensive silent behavior (B*_*member*_* = 0.64, *t_*member*_* = 20.53, *p* < 0.001; B*_*comunity*_* = 0.64, *t_*comunity*_* = 17.32, *p* < 0.001). Also, proactive personality showed a significant negative predictive effect on defensive silent behavior (B = –0.58, *t* = –8.03, *p* < 0.001), and defensive silent behavior had a significant negative predictive effect on member performance and community performance (B*_*member*_* = –0.07, *t_*member*_* = –4.36, *p* < 0.001; B*_*comunity*_* = –0.06, *t_*comunity*_* = –3.15, *p* < 0.01). However, when prosocial silent behavior acted as the mediating variable, although the results showed that proactive personality significantly positively affected member performance and community performance (B*_*member*_* = 0.68, *t_*member*_* = 22.72, *p* < 0.001; B*_*comunity*_* = 0.67, *t_*comunity*_* = 19.05, *p* < 0.001), and prosocial silent behavior had a significant positive effect on member performance and community performance (B*_*member*_* = 0.11, *t_*member*_* = 22.72, *p* < 0.001; B*_*comunity*_* = 0.12, *t_*comunity*_* = 3.79, *p* < 0.001), the effect of proactive personality on prosocial silent behavior was not significant (B = 0.05, *t* = 1.11, *p* > 0.05). This suggests that proactive personality does not predict the operation performance of online communities by controlling for the mediating effect of prosocial silent behavior. In particular, as shown in Table 6, in the test for proactive personality’s effect on the operation performance of online communities mediated by silent behavior, the upper and lower range of the bootstrap 95% confidence interval included 0, while neither of the bootstrap 95% confidence intervals included0 when the effect was mediated by acquiescent silent behavior or defensive silent behavior. This suggests that proactive personality not only directly affects member performance and community performance, but also indirectly through the mediating role of acquiescent and defensive silent behaviors. Thus, H6a and H6b were supported.

**TABLE 5:** Results of bootstrapping for the mediating effect.

Regression equation (*N* = 643)		SEM			Coefficient significance	
		
Outcome variable	Predictor variable	*R*	*R* ^2^	*F* (df)	B	t
Member performance	Proactive personality	0.67	0.44	511.15***	0.69	22.61***
Acquiescent silent behavior	Proactive personality	0.44	0.19	150.24***	–0.72	–12.26***
Member performance	Acquiescent silent behavior	0.68	0.47	280.53***	–0.11	–5.31***
	Proactive personality				0.61	18.46***
Defensive silent behavior		0.30	0.09	64.52***		
	Proactive personality				–0.58	–8.03***
Member performance		0.68	0.46	272.18***		
	Defensive silent behavior				–0.07	–4.35***
	Proactive personality				0.64	20.53***
Prosocial silent behavior		0.04	0.00	1.24***		
	Proactive personality				0.05	1.11
Member performance		0.68	0.46	272.59***		
	Prosocial silent behavior				0.11	22.72***
	Proactive personality				0.68	22.72***
Community performance	Proactive personality	0.60	0.36	362.35***	0.68	19.04***
Acquiescent silent behavior	Proactive personality	0.44	0.19	150.24***	–0.72	–12.26***
Community performance	Acquiescent silent behavior	0.61	0.37	187.16***	–0.07	–2.83**
	Proactive personality				0.63	15.99***
Prosocial silent behavior	Proactive personality	0.04	0.00	1.24***	0.05	1.11
Community performance	Prosocial silent behavior	0.61	0.38	192.14***	0.12	3.79***
	Proactive personality				0.67	19.05***
Defensive silent behavior	Proactive personality	0.30	0.09	64.52***	–0.58	–8.03***
Community performance	Defensive silent behavior	0.61	0.37	188.65***	–0.06	–3.15**
	Proactive personality				0.64	17.32***

***p < 0.001; **p < 0.01.

**TABLE 6:** Total effect, direct effect and mediating effect.

Paths	Effect	SE	LL95%CI	UL95%CI
PP—ASB—MP Total effect	0.69	0.03	0.63	0.74
Direct effect	0.61	0.03	0.54	0.67
Mediating effect PP—DSB—MP	0.08	0.02	0.42	0.11
Total effect	0.69	0.03	0.63	0.74
Direct effect	0.64	0.03	0.58	0.71
Mediating effect	0.04	0.01	0.02	0.07
PP—PSB—MP				
Total effect	0.69	0.03	0.63	0.74
Direct effect	0.68	0.03	0.62	0.74
Mediating effect	0.01	0.01	–0.01	0.02
PP—ASB—CP Total effect	0.68	0.04	0.61	0.75
Direct effect	0.63	0.04	0.55	0.71
Mediating effect	0.05	0.02	0.01	0.09
PP—DSB—CP				
Total effect	0.68	0.04	0.61	0.75
Direct effect	0.64	0.04	0.57	0.71
Mediating effect	0.04	0.01	0.01	0.06
PP—PSB—CP				
Total effect	0.68	0.04	0.61	0.75
Direct effect	0.67	0.04	0.60	0.74
Mediating effect	0.01	0.01	–0.01	0.02

CP, Community Performance; MP, Member Performance; PP, Proactive Personality; ASB, Acquiescent Silent Behavior; DSB, Defensive Silent Behavior; PSB, Prosocial Silent Behavior.

### Test for the moderating effect

In addition, we tested the moderating effect of online community identification and the results showed that evaluative online community identification negatively moderated the effect of prosocial silent behavior on member performance. H7 and H8 were partially supported. The results are presented in Table 7.

**TABLE 7:** The moderating effect of online community identification.

Regression equation (*N* = 643)			SEM		Coefficient significance	
			
Outcome variable	Predictor variable	*R*	*R* ^2^	*F* (df)	B	t
Member performance	Prosocial silent behavior	0.66	0.43	160.88***	0.589	2.87**
	Evaluative identification Prosocial silent behavior × Evaluative identification				1.09 –0.12	2.88*** –2.53**

***p < 0.001, **p < 0.01.

### Test for multi-collinearity

In this study, while conducting multiple regression analysis, we also pay attention to the multi-collinearity test of regression models, mainly focusing on Tolerance and Variance inflation factor (VIF) values. Rockwell (1975) thought that when Tolerance<0.1 or VIF > 10, there may have serious multicollinearity among independent variables. The test results of each regression model in this study show that the Tolerance value is between 0.43 and 1, and the VIF value is between 1 and 2.34, indicating that the regression analysis results are affected by the autocorrelation among independent variables in an acceptable range.

## Discussion and conclusion

### Results and discussion

This paper focuses on the relationship among online community members’ proactive personality in the real world, silent behavior, and operational performance of the online community, and explores the influencing factors and consequences of silent behavior in virtual communities different from organizations. Through the investigation and analysis of 643 online community members, the results show that:

(1) Proactive personality positively affects the operation performance of online communities. In other words, members’ proactivity can help improve the operation performance of an online community. Current research has mainly verified the relationship between proactive personality and individual performance, but few existing studies have addressed the relationship between proactive personality and group performance. This paper demonstrates that proactive personality as a positive self-driving force can be applied to improve the operation performance of an online community. These findings deepen and expand the theoretical study and understanding of proactive personality.

That being said, this research also verifies the inhibiting effect of proactive personality on silent behavior, thus, broadening the research on the antecedent variables of silent behavior and providing a new perspective and feasible path for reducing silent behavior within communities. Specifically, proactivity has a negative effect on acquiescent silent behavior and defensive silent behavior, and the higher the level of proactivity of online community members, the less acquiescent silent behavior and defensive silent behavior they exhibit, and this behavioral tendency of online community members has a positive impact on the improvement of the operation performance of online communities.

(2) The acquiescent and defensive silent behavior of online community members has a negative impact on the operation performance of an online community, but prosocial silent behavior has a significant positive impact on the operation performance of an online community. This suggests that silent behavior in online communities does not only have negative effects but can also exert a positive influence on the development of a community. This validates the conjecture proposed in this paper that silence is a double-edged sword. Thus, the findings of this study make a significant contribution to research on different types of silence, especially the impact of prosocial silent behavior.

(3) The acquiescent and defensive silent behavior of online community members has a significant mediating effect between proactive personality and the operation performance of online communities, while the mediating effect of prosocial silent behavior is not significant. That is to say, the level of the proactive personality of community members can affect their silent behavior by mainly influencing acquiescent and defensive silent behavior that is detrimental to the improvement of the operation performance of online communities. In turn, this influences the operation performance of online communities. Further, community members with higher proactivity display less acquiescent and defensive silent behavior. In addition, they will engage in more organizational citizenship behaviors, such as actively speaking up and participating in community activities, which will have a positive effect on the improvement of the operation performance of online communities.

(4) Online community identification moderates the effect of silent behavior on the operation performance of online communities. In other words, the higher the level of members’ online community identification, the less negative effect of silent behavior on the operation performance of online communities. Existing studies on the silent behavior of community members focus mainly on the constitutive dimensions of silent behavior, such as acquiescent silence, defensive silence, and prosocial silence, while few studies have explored the impact of silent behavior on the operation performance of an online community. This study selected the most common types of silent behavior, namely acquiescent silence, defensive silence, and prosocial silence, and added online community identification to the relationship between silent behavior and the operation performance of online communities. These ideas were based on social identity theory and used to investigate not only the direct impact of silent behavior on operation performance of online communities but also the moderating role of online community identification. The results validated the moderating role of online community identification, as members with high online community identification exhibit less silent behavior and facilitate the improvement of the operation performance of an online community. Moreover, because members with high online community identification are aligned with a community, members believe that they are representative of the community and that the community’s self-esteem represents their self-esteem. Therefore, to enhance the interests of the community, community members will reduce silent behaviors that are harmful to the community’s operation performance.

### Conclusion

The phenomenon of silent behavior in online communities is not only the focus of attention in the commercial operation of online communities but also the key to the operation of online communities. Based on the relevant theories in the field of organizational behavior, this paper systematically analyzes whether and how the silent behavior of online community members is affected by proactive personality in the real world and explores the impact of different dimensions of silent behavior on the operation performance of the online community. The results of this paper verify the double-edged sword effect of online community members’ silence behavior, further verify the impact of real-world proactive personality on online community members’ behavior, and the regulatory effect of online community identity on the relationship between silence behavior and operational performance, which enriches the relevant research on online community members’ behavior.

Firstly, this study expands the boundaries of the traditional theory of organizational silence behavior in the context of online communities, further analyzes the influencing factors and impacts of silence behavior in the virtual world and provides theoretical guidance for the online and offline integration in the organizational field and the emergence of the concept of metaverse. This study creatively explores the relationship between proactive personality and operation performance of online communities. While current research focuses on the relationship between proactive personality and individual performance, this study broadens the mechanism of the effect of proactive personality. In addition, most of the existing literature on operation performance focuses on the individual level and few studies examine the operation performance of online communities at the group level. This study finds that proactive personality is an important antecedent to the operation performance of an online community and is significant for ameliorating theories related to the operation performance of online communities. Secondly, this study classifies silence in online communities into three dimensions. By referencing organizational silence and investigating the negative and positive effects of silent behavior, respectively, this research verifies that silence is a double-edged sword. In addition, the research conducted an exploratory study on the positive effects of silence in the field of silence in online communities, thus enriching the related literature. Further, this paper is novel as it verifies the mediating role of silence between proactive personality and the operation performance of online communities, therefore, piloting the exploration of the relationship between these three variables and providing new ideas for research in related fields. Finally, using social identity theory, this study explores the moderating role of online community identification in the mechanism of silent behavior’s impact on the operation performance of online communities. As a result, these findings enrich and complement existing theoretical research on the consequences of organizational silence.

In addition, this study contributes to the theory of the relationship between proactive personality and silent behavior and analyzed the impact of individual characteristics in the real world on organizational behavior in the virtual world. Regarding the operation of online communities, community operators focus on motivating silent members to actively participate in communities to increase community activity. This paper finds that the higher the level of community members’ proactive personality, the more silent behavior is reduced, and the greater the positive impact on the operation performance of an online community.

At last, this study further analyzes the role of community identification in this process and believes that online community identification also plays a positive moderating role in relationship between proactive personality and the operation performance of online communities. This paper is novel as it verifies the mediating role of silence between proactive personality and the operation performance of online communities, therefore, piloting the exploration of the relationship between these three variables and providing new ideas for research in related fields. Finally, using social identity theory, this study explores the moderating role of online community identification in the mechanism of silent behavior’s impact on the operation performance of online communities.

Consequently, this research proposes several feasible insights for online community operators to improve operation performance. Firstly, research has indicated that a proactive personality is dynamic. Tornau and Frese (2013) showed that a proactive personality is associated with good job control and job support. In addition, Li et al. (2014) proposed that job demand, and a sense of job control can influence the emergence and development of a proactive personality. Specifically, a large volume of job demands can provide employees with opportunities to express their high proactivity and further contribute to the development of an individual’s proactivity. Therefore, community operators need to include active support and feedback in the participation of community members. In addition, they can increase the frequency of internal activities, such as posting challenging or interesting activities, to stimulate community members’ high proactivity and provide a good environment for community members to demonstrate active organizational citizenship behaviors, to improve the operation performance of the online community. For some communities, one might strictly control the requirements for membership to the community and only grant membership to individuals with high proactivity. This should create an active online community from the start and help to maintain high operation performance. Secondly, membership status can be distributed to community members. For example, the membership application implemented by the Douban-group developed group identification, thus encouraging active participation in online and offline activities organized by the community. This strengthened the connection between community members and increased group loyalty. Therefore, this approach can provide positive and timely feedback, enhance the self-worth of community members, and thus reduce the silent behavior of community members, thereby improving the performance of online communities. Thirdly, community operators should be aware that silent behavior is a form of self-protection. As a result, operators should pay attention to maintaining a harmonious community environment, creating an atmosphere that encourages active speaking, and removing hostile members. Finally, cash incentives and lotteries can be used to increase community members’ willingness to actively participate, thus, reducing their defensive silent behavior and improving the overall operation performance of online communities.

### Limitations and future research

There are relatively few studies that use social identification to analyze the impact of proactive personality and silent behavior on the operation performance of an online community. This paper has contributed exploratory research on this issue. However, the following limitations still exist. Firstly, the research was based on employee silence in organizational silence to classify the dimensions of silent behavior in online communities, and the applicability of the existing scales to online communities remains to be tested, especially with regards to prosocial silence. Subsequent studies can extract different dimensions of silent behavior in online communities or focus on the localization of silent behavior in online communities. Secondly, this paper did not take into consideration the influence of virtual roles and virtual personalities of online community members on silent behavior, and only considered the influence of proactive personality in the real world. The influence of virtual roles and virtual personalities on silent behavior needs to be further explored in the future.

## Data availability statement

The raw data supporting the conclusions of this article will be made available by the authors, without undue reservation.

## Author contributions

XP: conceptualization, formal analysis, supervision, and funding acquisition. FL: methodology. FL and XX: software. XP and FL: validation. JG and FL: investigation. WZ, JG, and FL: data curation. XP and WZ: writing—original draft preparation. XX: writing—review and editing. FL and AW: visualization. XX and AW: project administration and resources. All authors contributed to the article and approved the submitted version.
